# EQARO-ECS: Efficient Quantum ARO-Based Edge Computing and SDN Routing Protocol for IoT Communication to Avoid Desertification

**DOI:** 10.3390/s26030824

**Published:** 2026-01-26

**Authors:** Thair A. Al-Janabi, Hamed S. Al-Raweshidy, Muthana Zouri

**Affiliations:** 1 College of Electrical and Electronic Technical Engineering, Middle Technical University, Baghdad 10047, Iraq; 2 College of Engineering, Brunel University London, London UB8 3PH, UK; hamed.al-raweshidy@brunel.ac.uk; 3 Department of Computer Science, Toronto Metropolitan University, Toronto, ON M5B 2K3, Canada; mzouri@torontomu.ca

**Keywords:** ARO, desertification, edge computing, IoT, network lifetime, objective function, quantum gates, SDN, WSN

## Abstract

Desertification is the impoverishment of fertile land, caused by various factors and environmental effects, such as temperature and humidity. An appropriate Internet of Things (IoT) architecture, routing algorithms based on artificial intelligence (AI), and emerging technologies are essential to monitor and avoid desertification. However, the classical AI algorithms usually suffer from falling into local optimum issues and consuming more energy. This research proposed an improved multi-objective routing protocol, namely, the efficient quantum (EQ) artificial rabbit optimisation (ARO) based on edge computing (EC) and a software-defined network (SDN) concept (EQARO-ECS), which provides the best cluster table for the IoT network to avoid desertification. The methodology of the proposed EQARO-ECS protocol reduces energy consumption and improves data analysis speed by deploying new technologies, such as the Cloud, SDN, EC, and quantum technique-based ARO. This protocol increases the data analysis speed because of the suggested iterated quantum gates with the ARO, which can rapidly penetrate from the local to the global optimum. The protocol avoids desertification because of a new effective objective function that considers energy consumption, communication cost, and desertification parameters. The simulation results established that the suggested EQARO-ECS procedure increases accuracy and improves network lifetime by reducing energy depletion compared to other algorithms.

## 1. Introduction

The IoT refers to the physical connection of any nodes to the Internet. A wireless sensor network (WSN) is a main structure for IoT networking because it undertakes the mission of monitoring, gathering, and uploading data. However, in the traditional IoT networks, core processing is implemented by centralised servers that may be located far away from the associated nodes in the Cloud. Therefore, EC must be involved with the IoT architecture in which distributed processing brings data storage and calculation closer to the sources of data [1,2,3,4,5]. By analytically accomplishing calculations and computation at the EC, the communications bandwidth required between the control system and sensors at a centre is reduced. Pushing computation, calculation, and control near devices has numerous benefits, such as lowering transmission cost, latency, and consequent traffic, while enhancing performance, security, and reliability [6,7].

The two trendy paradigms of the Cloud and EC are essential for data processing and making decisions. However, each occupies different duties and cannot replace the other [8]. Cloud computing is useful for no-time-triggered data processing, whereas EC is useful for time-essential data processing. The Cloud framework supports inventors to easily manage, deploy, and create their applications. By scaling the evolution of the applications, the Cloud acts as an application data platform, which is associated with millions of end-user communications, and so on [6,9]. This framework can conduct analytics, store huge amounts of information, and create powerful visualisations. The EC paradigm is out of a centralised data centre, where it makes decisions using local servers and resources instead of sending the data to the Cloud. Generally, EC implements software, analyses, and process data closer to the end-user [8].

AI has also played a significant role in the past few years in relation to numerous issues [10,11,12]. Its main component is the optimisation of any problem and finding the best solution for such problem by minimising or maximising the optimal solution [13]. An efficient technique, called the quantum parallel technique, has become indispensable due to its ability to improve the accuracy of the solutions with the minimum required time and population size compared to other techniques [14]. Where measurements from these networks can be delivered in qubits instead of binary bits, and the best solution is kept in quantum computers without losing any of its characteristics, and then transferred within the available quantum networks [13].

Recent AI techniques, such as genetic algorithm (GA) [15,16], simulated annealing (SA) [17,18], and particle swarm optimisation (PSO) [19,20,21,22], have been widely used to control WSNs to improve and enhance the accuracy of the solution. However, these techniques continue to suffer from issues such as the consumption of extra energy, requiring additional control nodes, requiring location calculation, and being stuck in the local optimum. In comparison to other traditional low-energy adaptive clustering hierarchies (LEACHs) and PSO, the quantum technique is varied. Local optimal resolution passes over and reaches the global optimal resolution by employing the effect of quantum parallelism. Therefore, it can deliver higher performance solutions than other traditional techniques.

Some available state-of-the-art clustering algorithms only consider residual energy and cluster distances between nodes. They ignore other important parameters, such as desertification avoidance. The area of deserts and dryness increases due to human behaviour, such as causing fires in the desert or dry areas due to carelessness. Monitoring these areas using the IoT for collecting data and early detection of temperature changes or humidity levels can stop or avoid desertification [23]. Weather parameters, such as humidity and temperature, affect the performance of WSN. Therefore, an efficient multi-objective function based on energy, communication cost, and environment is developed. This study proposed an improved multi-objective routing protocol, namely, efficient quantum ARO routing protocol based on EC and the SDN concept (EQARO-ECS), which controls IoT devices and avoids desertification. The proposed protocol architecture includes various emerging technologies, which are the Cloud, EC, SDN, ARO [12], and quantum computing, to provide energy-efficient techniques suitable for desertification areas. To identify the best solution, the proposed protocol considers two essential directions, which are presented below.

**Figure 1:**
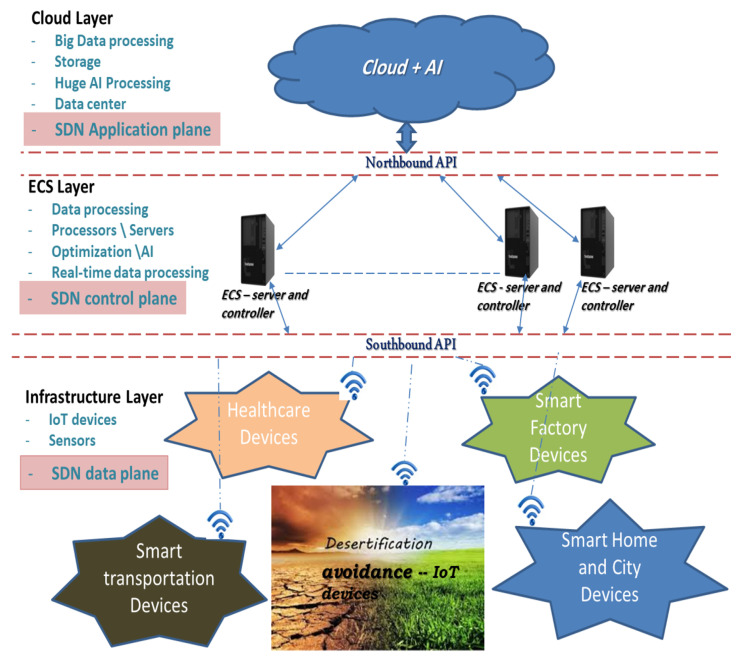
The proposed EQARO-ECS Architecture.

The first direction is the consideration of the objective function, in which the key issue with the development and IoT technologies is the sensor energy restriction due to the absence of stationary power sources [23]. Desert enlargement has become one of the main problems in dry areas for many reasons, such as increasing wildfires and temperatures. Therefore, the establishment of IoT technologies in such areas can improve and accelerate environmental observation by transmitting information, such as temperature, smoke, fire, and humidity, to the base station (BS) and controller to make quick decisions for such areas [24]. The other direction, the merging of AI techniques in IoT routing protocols, has the advantages of offering the best solution with lower computation complexity, but still has the issue of falling into the local optimum. Accordingly, this paper merges the latest optimisation technologies, which are the quantum concept and gates, to improve the performance of ARO, and to gain the best solutions. The major contributions of the proposed EQARO-ECS can be summarised as follows:

1. A new clustering technique-based ARO is deliberated. However, ARO still has some limitations as a research gap, such as easily falling into local optima, long processing time, and weak exploitation capability. Thus, the proposed technique merges quantum computing with ARO (QARO) to improve the speed and accuracy of the solution and to identify the best set of cluster nodes.
2. A strong objective function for the QARO is developed to cover the research gap of providing the best clusters used for quickly processing the sensed information. This objective function provided the best solution by considering the costs of three factors. These factors are the communication between nodes, energy consumption, and the diversification avoidance costs that have not been covered by previous research, such as humidity and temperature. Thus, this objective function can prolong network lifetime to avoid the desertification.
3. A suitable architecture, based on emerging technologies, such as EC, Cloud computing, AI, and quantum computing for IoT networks, has been developed (See Figure 1).
4. An innovative, efficient routing protocol named EQARO-ECS is suggested to enhance the performance of the proposed clustering protocol by offering an accurate and best solution of the clusters table (CT) for the IoT networking. Finding an accurate solution for the best clustering is one of the main research gaps in WSN routing protocols. This accurate solution is obtained by integrating quantum mechanics with various quantum probability amplitudes to recover the best solution.
5. An improvement on the quantum mechanics of finding the optimal CT is provided. This improvement covers a research gap by updating the quantum values of the amplitude probability multiple times. This is implemented by proposing a rotated quantum gate and an iterated quantum T-gate. In this case, if the operators fail to present population diversity, the proposed iterated T-gate offers different solutions instead of the first few solutions that are temporarily optimal. The proposed iterated T-gate reduces the probability of falling into a local optimum dilemma because of the utilisation of a large scale of high probability.
6. The results with comprehensive simulations are accompanied and compared to other techniques to validate the effectiveness of the suggested EQARO-ECS routing protocol.

The remainder of this research is structured as follows: In Section 2, a selection of related research by other scientists is discussed. The proposed work is divided into two categories: clustering-based classical algorithms and clustering-based AI and emerging technologies. Then, the proposed EQARO-ECS network architecture is clarified in Section 3. Then, the proposed objective function, methodology, measurement calculation, EQARO-ECS gates, and radio model of the proposed protocol are presented in Section 4, Section 5, Section 6, Section 7 and Section 8, respectively. Section 9 outlines the comprehensive simulation and results. Finally, this study is summarised by the conclusion and commentary on potential future work in Section 10.

## 2. Literature Review

To understand the relevance of the suggested technique, it is important to examine some well-known state-of-the-art techniques, such as EC, bio-inspired optimisation, quantum techniques, and some other inspiring applications. Clustering techniques have been broadly developed for the performance enhancement of IoT networking. However, to understand the work presented by EQARO-ECS, the literature review in this section is organised into two categories. The first category introduces the classical clustering algorithms, while the other describes those algorithms based on optimisation, meta-heuristic, and emerging technologies, such as techniques based on quantum algorithms (see Table 1).

### 2.1. Classical Clustering Algorithms

LEACH [25] is a single-hop-based probability method. The main measuring unit of this methodology is rounds. During each round, each device has a probability (*p*) of being a cluster head (CH). Accordingly, the CHs are elected based on their *p*-value. All CHs broadcast an announcement message to the adjacent devices. The non-CH devices transmit their data by electing a CH with the highest signal power.

Many similar approaches have been published with a few differences, such as the centralised LEACH (CLEACH) approach developed by Heinzelman et al. [26], stable election protocol (SEP) [27], threshold stable election protocol (TSEP) [28], and hybrid energy-efficient distributed clustering (HEED) [29]. More details and other approaches are presented in [46].

The authors in [30] suggest a methodology for distinguishing forest fires by including an element for controlling nodes remotely. The GSM unit is used for generating and sending an alert. In addition, the data aggregation process is performed using the Arduino board and then remotely transferred to a sink or a BS.

Another work, presented by Wang et al. [31], offered an advanced power-efficient gathering in sensor information systems (EPEGASIS) methodology to decrease the hotspot difficulty. The proposed methodology includes two phases. During the first phase, three main operations are implemented. These operations define the best distance cost to decrease energy depletion, localise the threshold to improve the lifetime of the vanishing sensors, and utilise mobile sink machinery to implement balancing for the energy depletion among the devices. The operation during the second phase is locating the communication distances among sensors and the BS. The simulation results of the presented method revealed that the suggested algorithm has an enhanced performance in comparison to other techniques in raising the overall network lifetime and minimising latency and power depletion in the network.

The authors in [32] deliberated on the improvement of using WSNs to recognise the fire sources in Indonesian forests. In this protocol, the WSN nodes are used for aggregating the environmental information and monitoring and recording any variations. The information is then sent as a report to the data centre for analysis and for making decisions. The sensors are fixed in numerous locations, especially where a fire has previously occurred, and another probable fire site is expected. In this work, an analytical model was used to identify the number of sensors required in the forested area.

All the above work used traditional techniques. In contrast, the work presented in this paper implements AI and emerging techniques to obtain the best sets of clusters.

### 2.2. Clustering-Based AI and Emerging Technologies

Numerous AI-driven emerging approaches for IoT routing algorithms have been provided, such as a reinforcement learning (RL)-based routing protocol for WSN [33,34,35], in which the AI works on dynamically learning the best paths, following some network circumstances [47]. Another algorithm is federated learning (FL) [36], which trains the model locally to improve efficiency and security without sharing any row data. Other bio-inspired techniques include ant colony optimisation [37,38]. Future search algorithm [5], WOA [39], or GAs [15,16], all improved routing protocol efficiency for the IoT.

Researchers in [40] offered an energy-aware routing algorithm for cluster formulation based on density (EA-DBCRP). This protocol, according to the collected WSNs data, formulates CT and distributes the load among the nodes in the network. As a result, the energy consumption is balanced among all nodes in the network, extending the lifetime of the network.

In [41], the authors presented a technique for forest fire discovery by developing an environmental technique for associating fault-tolerant routing algorithms that identify network reply time to an incident and network lifetime, taking into consideration network attributes. To investigate this technique, the examiners implemented advanced ant-based QoS routing technology in a heterogeneous WSN, called EAQHSeN, and a multi-level fault-tolerant routing technique, disconnected heterogeneous routing technology, called HDMRP.

However, all the above AI algorithms still have some limitations on processing speed and the exploration or exploitation stages. Therefore, this study employed ARO with a quantum technique to improve the shortcomings of the above algorithms. The researchers attempted to utilise the quantum principle or specific related constraints of the quantum procedure to recommend efficient methods to enhance the performance of the WSNs.

For instance, in [42], the authors offered the utilisation of a quantum genetic algorithm (QGA) to select routes among nodes and create connections for the purpose of packet exchanging. The main concept was to combine both the GA and quantum theory, using qubits to offer new genes that are normally applied to decide the best solution. The results showed an improvement in energy consumption and good network connectivity. The authors in [16,43] also studied the implementation of quantum theory to enhance the performance of GA and evolutionary techniques and attain capable cluster construction.

The authors in [44] proposed the quantum-inspired particle swarm optimisation for energy-efficient clustering (QPSOEEC) protocol, which uses a quantum-based technique for cluster formulation using a PSO. In this research, the CH selection process was implemented using the PSO technique, while quantum computing was utilised for position updating of the nodes. Another study was reported in [45], which utilised the QPSO to find the optimal set of clusters and enhance the correctness of the node position. However, this research is different because it considers a heterogeneous network, in which the nodes initially had various levels of energy and different communication capabilities.

With the above research improvements in computing optimisation and networking, various optimisation mechanisms have arisen. New AI algorithms continue to be presented to solve future complicated networking issues. This paper proposes the ARO with a quantum technique, based on an EC algorithm and supported by an efficient objective function to find the best set of clusters for IoT networking that avoids desertification factors.

## 3. The Proposed EQARO-ECS Network Architecture

### 3.1. Network Model

This study offers a desertification-based routing protocol for aggregating critical data useful for controlling IoT networks. The main challenge for WSN is resource restriction, especially relating to the energy power supply. Inadequate energy at these nodes is a major concern in prolonging the lifetime of the networks. This study suggests optimising energy depletion by considering energy consumption, communication cost, and desertification.

The main benefits of involving SDN in WSNs are energy efficiency, network reconfiguration, flexibility, scalability, network programmability, and intelligent routing decisions. In this case, the modifications of network management can be easily and flexibly implemented by uploading new applications on a device [48]. As seen in Figure 1, the SDN construction includes Application Programming Interfaces (APIs) that offer a working interface among the data, control and application planes. The southbound APIs exist between the data and control planes, while the northbound APIs are located among the control and application planes. The flows of control traffic from the controller to the devices in the infrastructure layer are implemented by the Southbound APIs.

The proposed architecture is based on the idea of SDN concept, combining the cloud, EC, and SDN technologies facilitate the decrease in traffic. This traffic is the amount of data that needs to be transmitted and processed in the Cloud so that these technologies can provide lower transmission costs and reduce latency. The SDN concept helps to improve the protocol performance by installing the SDN controller in the control plane, over the EC, to implement the necessary routing decisions. It is without any requirement for central Cloud processing. While the data plane located in the infrastructure layer (see Figure 1).

The merging of EC, SDN, and AI technologies in the network architecture to make cluster decisions has a direct effect on energy consumption, data processing, and network speed. To make the proposed EQARO-ECS routing protocol more realistic throughout the CT design and process, this research assumed that, first, the BS is centralised with no energy constrained; second, all the nodes are heterogeneous with two levels of initial energies; and third, all the nodes are stationary with fixed locations. This section describes how Cloud, EC, and SDN techniques have been founded and applied in the architecture of the proposed work. EC is a decentralised data processing technique that has the capability of enhancing the IoT performance, accessibility, and abilities.

Figure 1 shows a scheme for the proposed EQARO-ECS protocol. The figure is divided into three layers: The first layer is the infrastructure layer, which usually collects and detects data through small processing. In this layer, the nodes are collected and transmit data to the BS according to a predefined set of networking clusters by the controller. The second layer is edge computing with an SDN controller (ECS) layer. This layer is close to the infrastructure layer and is responsible for handling urgent and necessary data, which it processes in close to real time. The last layer is the Cloud layer, which is responsible for controlling multiple edges and for substantial storage and computations. The following subsections describe the three layers of the proposed work.

### 3.2. Cloud Layer

Cloud and edge technologies complement each other because the Cloud still has a significant role in the overall EC processing, such as providing a solution for network latency and congestion difficulties. Over the ECS layer, the proposed algorithm first determines if the data can be processed locally or if the operation is complex and needs to be moved and processed in the Cloud layer. AI processing is assumed to be implemented in the Cloud and the ECS Layers. For increasing efficiency, this paper suggested that there be fine-tuning of the computations in the ECS layer based on learning outcomes from processing big data in the Cloud Layer. For example, periodically synchronising algorithms and fine-tuning parameters between Cloud and the ECS Layers.

With normal operation, ECS also has to transfer critical or a portion of data to the Cloud layer to accomplish complicated decisions and/or savings. The proposed combination in this study of the Cloud and the ECS layers for IoT networking is efficient and a resourceful architecture.

### 3.3. Edge-Based SDN Concept Layer (ECS)

This layer, shown in Figure 1, involves EC servers and an SDN controller, which are distributed in a larger area in comparison to the Cloud layer. The EC-based SDN (ECS) layer provides lower latency, a lower amount of transmitted data, reduces the distance the data must travel, and reduces the resulting traffic. Thus, during ECS operations, servers are closer to the IoT nodes, which solves the speed and latency problems involved in processing in Cloud computing. In the proposed architecture, the ECS layer is a core processing unit for the entire network. This processing is performed by analysing the data received from the IoT devices in the infrastructure layer and then either transferring the complicated and huge information to the Cloud layer or responding to the infrastructure layer with optimal CT. The ECS layer involves the implementation of QARO with an efficient objective function, using the SDN controller over EC to find the optimal CT depending on the information collected from the devices. The analysed information is then forwarded to the infrastructure layer and transmitted to the Cloud layer for further processing and saving.

### 3.4. Infrastructure Layer

As shown in Figure 1, the infrastructure layer comprises many heterogeneous devices from small to large, such as mobile phones, temperature sensors, transportation, healthcare, smart homes, and factories. In this layer, the devices gather and capture the required data for making routing decisions or achieving the goal they are designed for. The location information must be involved in the data attained by the devices; otherwise, the observing information is insignificant. The devices send the data to the ECS or Cloud layers through the BS. However, the nodes in the infrastructure layer must still perform some processing, storage, or data analysis, which require energy resources but have the advantage of processing significant data in real time.

## 4. Objective Function

The proposed objective function is implemented by the SDN controller at the ECS layer. The controller calculates three essential factors, which are the residual energy, communication cost, and desertification avoidance factors, such as humidity and temperature. In each round, the average energy related to the live nodes is calculated, and then the nodes are candidates for being CHs with energy greater than the average in a matrix named $L_{CH}$. The SDN controller maximises the cost of the subsequent equations:

(1) $$f_{1}=\sum_{m=1}^{CH_{N}}\frac{\sum_{v=1}^{CH_{N}}Eng_{CH_{\left(m,v\right)}}}{\sum_{v=1}^{V}Eng_{Pop_{\left(m,v\right)}}}$$

(2) $$f_{2}=\sum_{m=1}^{CH_{N}}\frac{\sum_{v=1}^{V}dis\left(n_{v},BS\right)}{\sum_{v=1}^{N_{m}}dis\left(n_{\left(m,v\right)},CH_{m}\right)}+\frac{\sum_{v=1}^{V}dis\left(n_{v},BS\right)}{\sum_{m=1}^{CH_{N}}dis\left(CH_{m},BS\right)}$$

(3) $$f_{3}=\left\{\begin{array}{cc} |\frac{\frac{\sum_{m=1}^{CH_{N}}Temp_{CH_{m}}}{CH_{N}}}{Temp_{avg}}|+\frac{\frac{\sum_{m=1}^{CH_{N}}Hum_{CH_{m}}}{CH_{N}}}{Hum_{avg}}, & if\;C\leq Temp_{Dsrt}\;\&\\ & \;\;\;Hum_{Dsrt}\geq R\\ |\frac{Temp_{avg}}{\frac{\sum_{m=1}^{CH_{N}}Temp_{CH_{m}}}{CH_{N}}}|+\frac{Hum_{avg}}{\frac{\sum_{m=1}^{CH_{N}}Hum_{CH_{m}}}{CH_{N}}}, & Otherwise \end{array}\right.$$

where

(4) $$Temp_{Dsrt}=|Temp_{avg}-\frac{\sum_{m=1}^{CH_{N}}Temp_{CH_{m}}}{CH_{N}}|$$

(5) $$Hum_{Dsrt}=Hum_{avg}-\frac{\sum_{m=1}^{CH_{N}}Hum_{CH_{m}}}{CH_{N}}$$

(6) $$Temp_{avg}=\frac{\sum_{v=1}^{V}Temp_{\left(n_{v}\right)}}{V},$$

(7) $$Hum_{avg}=\frac{\sum_{v=1}^{V}Hum_{\left(n_{v}\right)}}{V},$$

(8) $$Cost=\omega f_{1}+\beta f_{2}+\tau f_{3}$$

where the coefficients for the energy, communication cost, and factors of desertification are represented as ω, β and τ, respectively, and are shown in Table 2. The parameters Pop and $CH_{N}$ denoted the populations presented in Equation (9) and number of CHs in Equation (11), respectively. C and R are selected to be 0 and 0.2, respectively and specify the range of nodes to be CHs with perfect environmental temperature and humidity.

Weather conditions, such as humidity and temperature, affect the performance of WSN. Therefore, an efficient multi-objective function based on energy, communication cost, and environment was developed. The first function $f_{1}$ selects the CHs collection with a higher energy level. The second function $f_{2}$ chooses cluster sets with the smallest interior and exterior communication costs, where the interior communication cost is the communication between CM and CH, and exterior communication is the communication among the CHs and the BS. Finally, function $f_{3}$ chooses the set of clusters with higher suitability to avoid desertification. This function is proposed to nominate nodes with moderate temperature ($Temp_{avg}$) and Humidity ($Hum_{avg}$) factors to be CHs. These two factors are used in defining the desertification temperature ($Temp_{Dsrt}$) and humidity ($Hum_{Dert}$) that consolidate the overall network to live, in average, for the same lifetime.

Subsequently, the SDN controller organises the data transmission schedule using a mechanism called time division multiple access (TDMA). Through this scheduler mechanism, all the CMs transmit their related ID, residual energy, and data to their connected CH throughout their identified time slot, while conserving energy by keeping their radio off during all other time slots.

## 5. The Methodology of the Proposed Model

**Figure 2:**
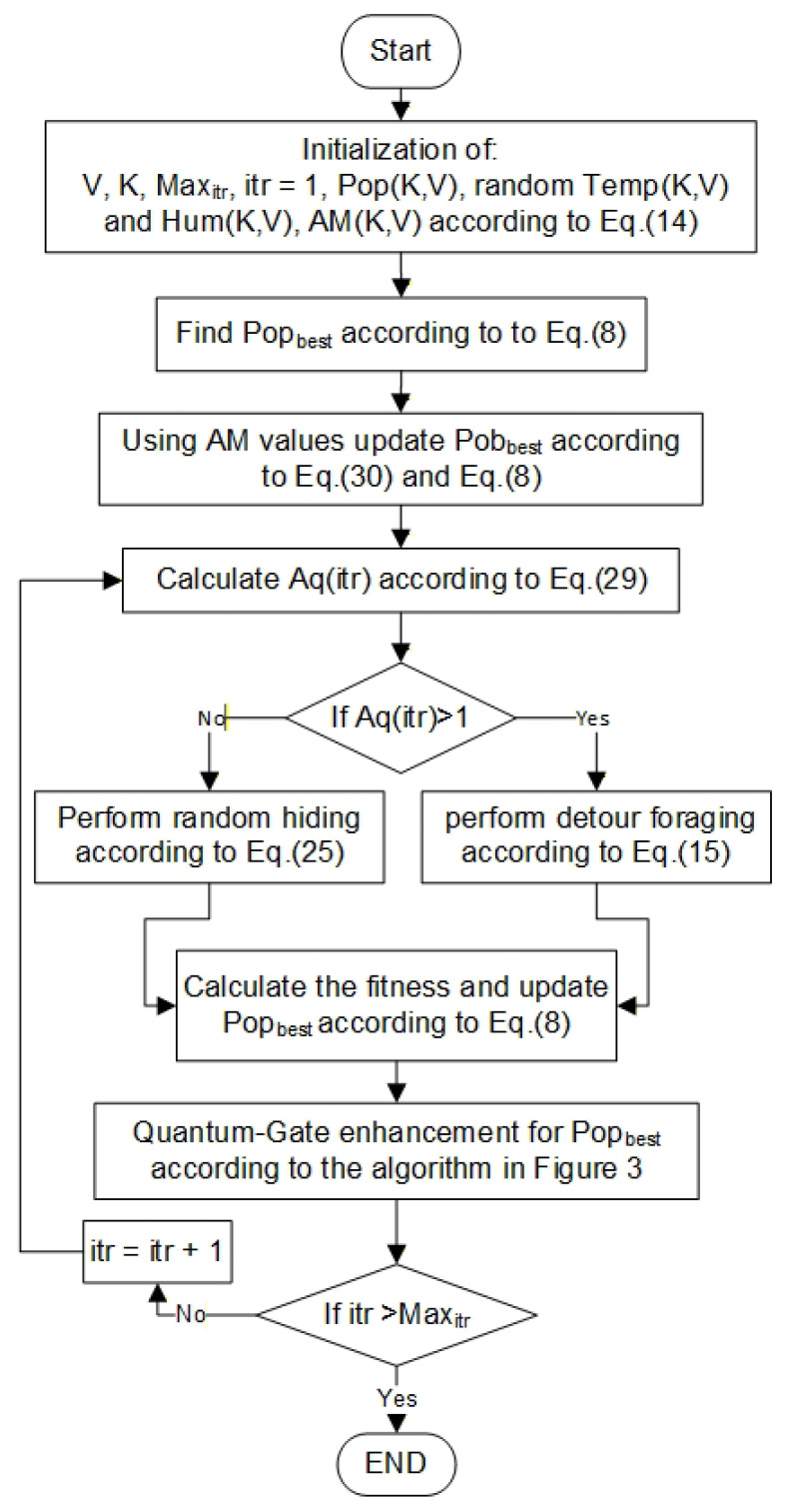
Flowchart for finding the best solution using the proposed EQARO-ECS.

The resiliency in the real world is considered as an essential criterion during the IoT protocol design. This paper aims to identify an efficient protocol responsible for providing a CT to prolong the IoT network lifetime in the desertification areas. This is because of the development of Equation (3) that attempts to choose the set of CHs with an average weather temperature. The architecture of this protocol exploits edge and Cloud resources in addition to the implementation of AI and quantum techniques by the SDN controller to identify the best solution. The methodology of EQARO-ECS, presented in Figure 2, is organised into many subsections, which are individual populations, quantum state and probability amplitude generation, measurement and fitness calculations, QARO implementation, and quantum gates. The cluster set construction is managed by the SDN controller at the ECS layer by creating the CT using the EQARO-ECS algorithm.

For the first stage, the SDN controller constructs the CT using only the information about the node coordinates. For the following stages (rounds), the SDN controller exploits the aggregated data regarding the temperature, humidity, and the nodes’ remaining energy and communication cost to find the best group of clusters. Then, the controller implements QARO to identify the CT, which possesses scheduling information and the clusters set, and transmits it to the IoT nodes in the infrastructure layer through the BS.

### 5.1. Individual Initialization

Initially, QARO-ECS randomly generates the value of each individual in a population matrix, called Pop, with a set of zeros and ones. The size of the generated matrix is K × V, where K represents the number of individuals to be tested in the network, and V represents the number of nodes in each individual and is equal to the number of nodes in the network. The matrix Pop can be represented by Equation (9):

(9) $$Pop=\left[\begin{array}{cccc} Pop_{1,1} & Pop_{1,2} & \ldots & Pop_{1,V}\\ \vdots & \vdots & \ddots & \vdots \\ Pop_{K-1,1} & Pop_{K-1,2} & \ldots & Pop_{K-1,n}\\ Pop_{K,1} & Pop_{K,2} & \ldots & Pop_{K,V} \end{array}\right].$$

### 5.2. Qubit State Identification and Generation

Unlike the classical computer that uses only zero or one binary states, the data representation with the quantum computer is represented by a qubit with various probability values. The value of the qubit can be similar to that of a classical computer with only zero or one states, or it can be a set of states by including different probability values, named superposition. Equation (10) shows a $2^{n}$ state kind that represents a qubit of size n:

(10) $$AM=\left(\begin{array}{ccccc} \left|\begin{array}{c} \alpha_{1}\\ \alpha_{1}^{\prime } \end{array}\right| & \left|\begin{array}{c} \alpha_{2}\\ \alpha_{2}^{\prime } \end{array}\right| & \ldots & \left|\begin{array}{c} \alpha_{V-1}\\ \alpha_{V-1}^{\prime } \end{array}\right| & \left|\begin{array}{c} \alpha_{V}\\ \alpha_{V}^{\prime } \end{array}\right| \end{array}\right).$$

Generally, the number of CHs ($CH_{N}$) for each $Pop_{K,V}$ can be calculated by Equation (11) as below:

(11) $$CH_{N}=\sum_{i=1}^{V}{Pop_{j,i}}_{\left(j:1,2,\ldots ,K\right)}.$$

Whereas the state of the quantum qubit can be represented by Equation (12):

(12) $$|\gamma \rangle =\alpha |0\rangle +\alpha^{\prime }|1\rangle$$

where α and $\alpha^{\prime }$ represent the amplitude probabilities for the quantum states 0 and 1, respectively. These probabilities have constraints presented in Equation (13):

(13) $${|\alpha \rangle }^{2}+{|\alpha^{\prime }\rangle }^{2}=1$$

where ${|\alpha \rangle }^{2}$ and $|\alpha^{\prime }\rangle^{2}$ signify the probability that a quantum state is perceived as a state zero and state one, respectively. Each individual in the matrix Pob is equipped with an amplitude probability $AM_{i}$ that can be generated according to the following pseudo-random number generator in Equation (14):

(14) $$AM_{v+1}=\frac{(AM_{v}\;\mathrm{mod}\;12)-1}{12}\left(v:1,2,\ldots ,V\right).$$

Equation (14) provides a set of pseudo-random numbers between [0, 1]. Initially, the seed value of $AM_{0}$ is a random number between [0, 12]. The benefit of applying such pseudo-random numbers is to raise the consistency of the preliminary AM matrix, thus earning a superior cluster set structure for the proposed EQARO-ECS.

### 5.3. ARO

Throughout this section, the idea and emulation behind the ARO technique are presented (see Algorithm 1). In the subsequent sections, the mathematical models and strategies for implementing the energy model for the proposed QARO are presented.
**Algorithm 1** ARO Algorithm. 1: Initialization of: 2: $Pop\leftarrow Pop_{1,1}\ldots Pop_{K,V}$ 3: itr←1 4: $Max_{itr}$ 5: Find $Pop_{best}$ according to Equation (8) 6: **while** $itr<Max_{itr}$ **do** 7:       **for** every Pop **do** 8:             Select the guide rabbits from Pob 9:             Calculate Aq(itr)10:             **if** Aq(itr) > 1 **then**11:                Update $Pop_{best}$ according to Equation (15)12:             **else**13:                Update $Pop_{best}$ according to Equation (25)14:             **end if**15:       **end for**16:       Update $Pop_{best}$ according to Equation (8)17: **end while**

### 5.4. ARO Mechanism

The ARO mechanism is inspired by the rabbit’s survival strategy, which is widely spread as a common Chinese phrase, ’rabbits do not eat the grass near their own nest’ [12].

This strategy allows the rabbits to find food easily over a large area because of the wide field of vision of rabbits. They focus on overhead scanning, so that they can easily find food over a large area. This foraging strategy prevents rabbits from eating the grass close to their rabbit holes and means that they always seek food far from their nests. Throughout the ARO technology, the detour foraging strategy was considered as the exploration phase.

To escape tracking by hunters or predators, rabbits make nests by digging many burrows near their nests and randomly selecting one as a haven from predators. This random hiding behaviour of rabbits is a survival strategy. Rabbits have many strategies to escape tracking by enemies, which raises the likelihood of their survival. These strategies are running fast, escaping in zigzag motion, stopping unexpectedly, running back, and turning around suddenly. The ARO also uses random hiding and a survival scheme as an exploitation phase. Based on the high number of the rabbits’ predators, the rabbits must escape danger by running fast and, based on their energy, having to adaptively change between random hiding and detour foraging [12].

### 5.5. Model and Algorithm of ARO

Two main strategies were employed by the ARO: foraging and hiding approaches. Transiting between those strategies according to their energy is termed the energy shrink strategy. These strategies are described in the next subsections.

#### 5.5.1. Detour Foraging (Exploration)

In detour foraging, rabbits leave the grass closest at hand and seek distantly for food. ARO uses a similar methodology, where the working area is divided into regions with grass and food. Each rabbit in the swarm has one region and *V* burrows. However, the rabbits usually visit other areas randomly for foraging and gaining adequate food.

The ARO’s detour foraging randomly updates each position towards the other areas in the swarm and adds a perturbation. The mathematical representation of the rabbit’s detour foraging is represented as follows [12]:

(15) $$\begin{array}{c} {\vec{vr}}_{i}\left(t+1\right)={\vec{Pop}}_{j}\left(t\right)+\Gamma \times \left({\vec{Pop}}_{i}\left(t\right)-{\vec{Pop}}_{j}\left(t\right)\right)\\ +round\left(0.5\left(0.5+\hbar_{1}\right)\right)n_{1},\\ i,j=1,\ldots ,K\;and\;j\neq i \end{array}$$

(16) Γ=Λ×cq

(17) $$\Lambda =\left(e-e^{{\left(\frac{t-1}{Max_{itr}}\right)}^{2}}\right)\sin\left(2\pi \hbar_{2}\right)$$

(18) $$c\left(v\right)=\left\{\begin{array}{cc} 1, & \mathrm{if}\;v==\mu (l)\\ \; & v=1,\ldots ,V\;and\;l=1,\ldots ,\lceil \hbar_{3}\times V\rceil \\ 0, & \mathrm{otherwise} \end{array}\right.$$

(19) μ=randperm(V)

(20) $$n_{1}≃K\left(0,1\right)$$

where $K,n_{1},\Lambda ,dq,(\hbar_{1},\hbar_{2}$ and $\hbar_{3})$ and $Max_{itr}$ are rabbit population size, standard normal distribution, movement step length when implementing the detour foraging, problem dimension, three random numbers in range (0,1) and maximum iteration number, respectively. The ${\vec{Pop}}_{j}\left(t\right)$ represents the *i*th rabbit location at time *t*, while ${\vec{vr}}_{i}\left(t+1\right)$ represents the *i*th rabbit probable location at time t+1.

#### 5.5.2. Random Hiding (Exploitation)

Like the rabbits’ behaviour of digging multiple burrows for hiding and survival, at each iteration, ARO usually creates *V* burrows beside each search space dimension and then randomly selects one of them for hiding to decrease the possibility of being preyed upon. The subsequent equation is agreed upon in this respect. The generation of the *j*th burrow for the *i*th rabbit is represented as the following [12]:

(21) $$\begin{array}{c} {\vec{br}}_{i,j}\left(t\right)={\vec{Pop}}_{i}\left(t\right)+Hq\times \mu \times {\vec{Pop}}_{i}\left(t\right),\\ i=1,\ldots ,K\;and\\ j=1,\ldots ,V \end{array}$$

(22) $$Hq=\frac{Max_{itr}-t+1}{Max_{itr}}\times \hbar_{4}$$

(23) $$n_{2}≃K\left(0,1\right)$$

(24) $$\mu \left(v\right)=\left\{\begin{array}{cc} 1, & \mathrm{if}\;v==j\\ \; & v=1,\ldots ,V\\ 0, & \mathrm{otherwise}. \end{array}\right.$$

From Equation (21), the *V* burrows are created in the vicinity of a rabbit alongside each dimension, while *H* is the hiding factor that is linearly reduced from 1 to $\frac{1}{Max_{itr}}$ [49]. For survival, rabbits have to discover a safe residence for hiding because they are regularly subjected to attack by predators. Thus, they randomly choose a burrow to avoid being caught. This random hiding approach can be mathematically modelled by the following equations:

(25) $$\begin{array}{c} {\vec{vr}}_{i}\left(t+1\right)={\vec{Pop}}_{i}\left(t\right)+\Gamma \times \left(\hbar_{4}\times {\vec{br}}_{i,r}\left(t\right)-{\vec{Pop}}_{i}\left(t\right)\right)\\ i=1,\ldots ,K \end{array}$$

(26) $$\mu_{r}\left(v\right)=\left\{\begin{array}{cc} 1, & \mathrm{if}\;v==\lceil \hbar_{5}\times V\rceil \\ \; & v=1,\ldots ,V\\ 0, & \mathrm{otherwise} \end{array}\right.$$

(27) $${\vec{br}}_{i,r}\left(t\right)={\vec{Pop}}_{i}\left(t\right)+Hq\times \mu_{r}\times {\vec{Pop}}_{i}\left(t\right),$$

where $\hbar_{4}$ and $\hbar_{5}$, which range in (0, 1), are two random numbers. While ${\vec{br}}_{i,r}$ signifies a hiding burrow that is randomly chosen from its *V* burrows. The *i*th search individual in Equation (25) modifies its location to the burrow locations that are randomly selected from its *V* burrows. After achieving both detour foraging and random hiding, the *i*th rabbit position is updated according to the following equation:

(28) $${\vec{Pop}}_{i}\left(t+1\right)=\left\{\begin{array}{cc} {\vec{Pop}}_{i}\left(t\right), & f\left({\vec{Pop}}_{i}\left(t\right)\right)\leq f\left({\vec{vr}}_{i}\left(t+1\right)\right)\\ {\vec{vr}}_{i}\left(t+1\right), & f\left({\vec{Pop}}_{i}\left(t\right)\right)>f\left({\vec{vr}}_{i}\left(t+1\right)\right) \end{array}\right..$$

This equation explains that whenever the new probable location of the *i*th rabbit is better than the present one, the rabbit leaves the present location and moves to the new location produced by Equation (15) or Equation (25).

### 5.6. Energy Shrink (Switch from Exploration to Exploitation)

In ARO, rabbits are permanently inclined to regularly accomplish detour foraging in the preliminary stage of repetitions and then regularly accomplish random hiding in the late stage of reiterations. Depending on the energy of the rabbit, the search mechanism shrinks steadily and moves between exploration and exploitation phases, following the energy factor Aq(t), which is defined by the following equation:

(29) $$Aq\left(t\right)=4\left(1-\frac{t}{Max_{itr}}\right)ln\frac{1}{\hbar }$$

where Aq(t) and *ℏ* represent an energy factor and a random number in the range (0, 1).

## 6. Measurement and Fitness Calculation

After initialisation and ARO implementation, measurement and fitness calculations are implemented. During the measurement process, the quantum matrix with AM probabilities must be measured according to certain criteria and function, in which each bit in the $AM_{th}$ is evaluated and measured as follows:

(30) $$Msu._{v}=\left\{\begin{array}{cc} 1, & var>|\alpha |\\ 0, & var\leq |\alpha^{\prime }| \end{array}\right.$$

where $Msu._{v}$ represents a binary solution for each (rabbit) for AM measurement. While var is a random number ∈[0,1]. The fitness function aims to identify the best solution ($Pop_{best}$) and to move all other individuals in the direction of that optimal solution. Each solution is evaluated according to Equation (8) and represents a CT with CHs and their connected CMs. The best solution with a high fitness value represents the optimal cluster set with low communication cost and high remaining energy, avoiding the desertification areas.

## 7. EQARO-ECS Quantum Gates

**Figure 3:**
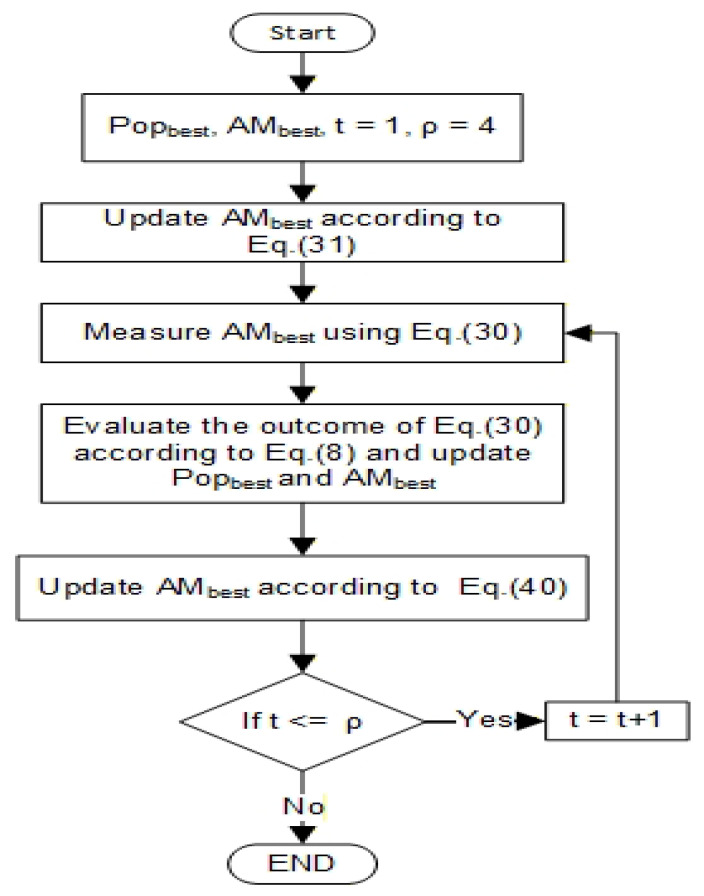
Flowchart for updating $Pop_{best}$ values using the proposed quantum gates to find a better solution.

To solve the clustering problems of WSNs, the proposed EQARO-ECS modifies the values of each qubit of individuals, rendering the best solution from the ARO operation. Two essential gates are proposed, which are the rotation gate and the iterated T-gate. However, for more space to discover the best and most accurate solution, an algorithm, named iterated T-gate, which iterates the T-gate in various space directions, has been suggested (see Figure 3).

### 7.1. Rotation Gate

In EQARO-ECS, the final CTs are identified by updating each qubit amplitude of individuals after obtaining the best amplitude solution ($AM_{best}$) using the ARO algorithm. The modifying principle is presented as follows [50]:

(31) $$\left[\begin{array}{c} \alpha_{i}update\\ \alpha_{i}^{\prime }update \end{array}\right]=\left[\begin{array}{cc} \cos(\vartheta_{i}) & -\sin(\vartheta_{i})\\ \sin(\vartheta_{i}) & \cos(\vartheta_{i}) \end{array}\right]\left[\begin{array}{c} \alpha_{i}\\ \alpha_{i}^{\prime } \end{array}\right]$$

where $\vartheta_{i}$ is the value of the updating phase used to modify the amplitude probability according to the following formula:

(32) $$\vartheta_{i}=\Delta \vartheta_{i}\times \delta \left(\alpha_{i},\alpha_{i}^{\prime }\right)$$

(33) $$\Delta \vartheta_{i}=5\exp^{\frac{-t}{T}}$$

where $\Delta \vartheta_{i},t$, and $Max_{itr}$ control the period’s rotation speed, the period’s sequence, and the maximum number of iterations, respectively. The formula $\delta (\alpha_{i},\alpha_{i}^{\prime })$ is identified as follows:

(34) $$\delta \left(\alpha_{i},\alpha_{i}^{\prime }\right)=\left(\frac{def_{ibest}}{def_{icurrent}}\right)\left(\ell_{ibest}-\ell_{icurrent}\right)$$

where

(35) $$def_{ibest}=\frac{\alpha_{ibest}^{\prime }}{\alpha_{ibest}}$$

(36) $$def_{icurrent}=\frac{\alpha_{icurrent}^{\prime }}{\alpha_{icurrent}}$$

(37) $$\ell_{ibest}=\arctan\left(\frac{\alpha_{ibest}^{\prime }}{\alpha_{ibest}}\right)$$

(38) $$\ell_{icurrent}=\arctan\left(\frac{\alpha_{icurrent}^{\prime }}{\alpha_{icurrent}}\right)$$

where $\alpha_{icurrent},\alpha_{icurrent}^{\prime },\alpha_{ibest}$, and $\alpha_{ibest}^{\prime }$ are the current and best probabilities of the *i*th qubit, respectively.

### 7.2. Iterated T-Gate

The T-gate is a member of a single-phase shift family that keeps the single state |0〉 and updates the |1〉 state with $\frac{\pi }{4}$ phase, according to the following phase matrix representation:

(39) $$T_{Gatematrix}=\left[\begin{array}{cc} 1 & 0\\ 0 & \exp^{\frac{i\pi }{4}} \end{array}\right]$$

(40) $$\left[\begin{array}{c} \alpha_{update}\\ \alpha_{update}^{\prime } \end{array}\right]=\left[\begin{array}{cc} 1 & 0\\ 0 & \exp^{\frac{\rho \pi }{4}} \end{array}\right]\left[\begin{array}{c} \alpha \\ \alpha^{\prime } \end{array}\right]$$

where ρ, seen in Table 2, is the rotation iteration number for the iterated T-gate proposed to be used in this paper, while ϑ is the phase gate equivalent to $\frac{\pi }{4}$. The value of the ρ is chosen to be four, such that a half-space investigation is performed. For further investigation, the ρ value can be upgraded up to eight. However, in this research, four iterations were chosen for less processing and complexity. This gate is equivalent to horizontally tracing or rotating around the y−axis, like the Bloch sphere method. However, the probability during this gate for measuring a |0〉 or |1〉 is higher due to the amplitude amplification. As shown in Figure 3, after each iteration, a measurement on each quibit was applied, using Equation (30), and then the fitness of the result, according to Equation (8). The value of $AM_{best}$ is to keep updating according to Equation (40).

### 7.3. Complexity Analysis and Overhead

LEACH and SEP techniques have lower analytical complexity compared to other meta-heuristic PSO and the proposed EQARO-ESC techniques that present significantly higher analytical necessities. Therefore, this section only considered the computation complexity for PSO and EQARO-ESC protocols. The complexity of PSO is equivalent to the ARO complexity and computed by identifying $Max_{itr},V$, and *K* and computed by Equation (41):

(41) $$PSO=ARO=Max_{Itr}\times V\times K.$$

Accordingly the ARO complexity can be represented by $O(Max_{Itr}\times V\times K)$. Whereas the computation complexity of the proposed EQARO-ESC primarily arises because of the ARO and the proposed iterated quantum gates and it can be conveyed by the following Equation (42):

(42) $$quantum_{Iterated}=Max_{Itr}\times \rho \times K\times V.$$

Hence, the overall complexity analysis of EQARO-ESC can be stated by Equation (43):

(43) $$EQAROESC_{Complexity}=Max_{Itr}\times V\times K\times \left(1+\rho \right).$$

Compared to PSO, the suggested EQROA-ESC mostly increases the complexity obtained from the iterated quantum machinist. However, the complexity analysis of the suggested algorithm is still in a satisfactory range, because (1+ρ) in Equation (43) is constant and can be ignored, then $EQAROESC_{Complexity}$ can be represented by $O(Max_{Itr}\times V\times K)$, which is equivalent to the complexity presented by PSO algorithm.

The transmission of control massages is considered as the core causes of the overhead during the CT formulation process. Therefore, the calculation of the overall overhead, for the classical clustering algorithm, i.e., LEACH algorithm, can be implemented by adding the energy spent by all devices for transmitting and receipting of three main control messages. These messages are advertisement (ADV) broadcast by the candidate CH, join–request (Join) transmitted by the nodes to join the appropriate CH, and TDMA (Schedule) to schedule the cluster nodes transmissions.

While the overall overhead generated by the proposed protocol based optimization algorithm can be calculated by adding only the energy spends by the ADV and Schedule control messages without the requirement for the Join messages, which makes the proposed EQARO-ECS protocol have lower overhead than others.

## 8. Radio Model

This study aimed to assess the average energy spent by a device, depending on various constraints, such as the energy dissipated during transmission, reception and data collection. The first-order analytical model presented by [26] evaluates the energy consumed by the proposed EQARO-ECS. Each device spends energy in data aggregation ($Eng_{DA}$), amplification ($Eng_{amp}$), reception ($Eng_{RX}$), transmission ($Eng_{TX}$), and desertification factors effects ($Eng_{Dsrt}$). To attain the specified ranks of signal-to-noise ratio for one bit sending over a distance (dis), the $Eng_{amp}$ is present as follows:

(44) $$Eng_{amp}=\left\{\begin{array}{cc} \xi_{FS}dis^{2},\; & if\;dis<dis_{0}\\ \xi_{TR}dis^{4},\; & if\;dis\geq dis_{0} \end{array}\right.$$

where $\xi_{FS}$, $\xi_{TR}$, and $dis_{0}$ are distinct in Table 2. However, the energy spent to transfer or collect lbits over dis is identified as follows:

(45) $$Eng_{TX}\left(l,dis\right)=lEng_{elec}+lEng_{amp}$$

(46) $$Eng_{RX}\left(l,dis\right)=lEng_{elec}$$

(47) $$Eng_{Dsrt}=Eng_{TX}\left(l,des\right)\left(\frac{Temp_{Dsrt}+Hum_{Dsrt}}{2}\right)$$

where $Eng_{elec}$ is the energy spent per bit for broadcasting or receiving. Likewise, all other radio factors are presented in Table 2.

## 9. Simulation Results

MATLAB 2014b was used throughout the implementation of the proposed protocol. A total of 100 heterogeneous nodes were randomly distributed over a 100 × 100 m^2^ area. The nodes are assumed to be heterogeneous to emulate the diversity of IoT devices. However, for simplicity, the network nodes are classified into two levels of heterogeneity, where 50% of the nodes had 0.5 Joules/s of energy and the others were supplied with a 1 Joules/s battery (similar to the node classification presented by [51]). The sink node was an unlimited power supply with no resource restrictions.

To prove the performance and correctness of the proposed EQARO-ECS, a comparison with the intelligent technique of PSO [22] and other classical techniques including LEACH [25] and SEP [27] were presented. However, the implementation of these techniques needed to dissipate higher energy to gain a global best solution or they could cause trapping in a local best solution issue. The concrete comprehension solution was to obtain the local best solution by choosing a very small probability with the application of a quantum technique.

**Figure 4:**
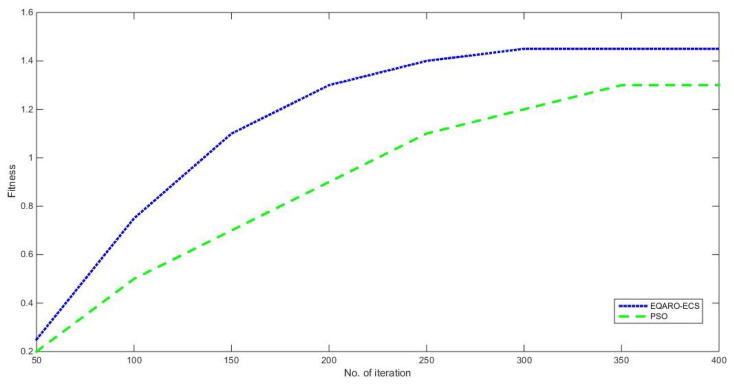
The convergence property of EQARO-ECS and PSO.

Figure 4 evaluates the convergence property by displaying the best value F(t) of EQARO-ECS and PSO versus the increasing number of iterations. It can be notice that PSO start to be stable with about 350 iterations, while it only take about 290 iterations for EQARO-ECS. This illustrates that EQARO-ECS has a quicker convergence proportion than PSO.

**Figure 5:**
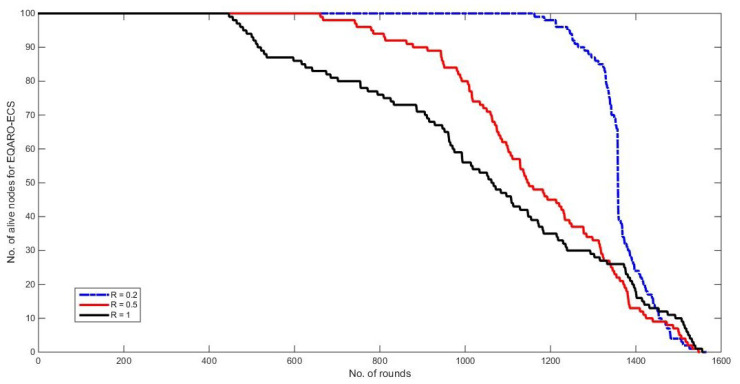
The fitness cost function (cost(t)) values between the proposed EQARO-ECS and PSO.

Figure 5 shows a comparison of the fitness cost function (cost(t)) values between the proposed EQARO-ECS with different R values. It can be noted from the figure that most of the network are live approximately for the same time especially when R = 0.2, this is because of the implementation of the overall average of Equations (6) and (7) that utilised by Equation (8). This was due to the involvement of critical factors, such as the association of quantum computing, edge technology, SDN, and efficient fitness function when determining the best solution.

**Figure 6:**
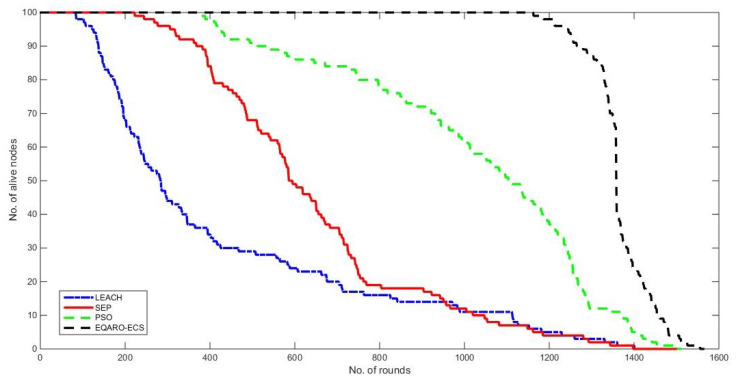
The network lifetime of EQARO-ECS, PSO, SEP, and LEACH.

Figure 6 demonstrates network lifetime. Where the lifetime in networking was considered as the number of nodes active over rounds (time) from the initiation of the communication till all nodes in the network were deactivated. In comparison to other algorithms, EQARO-ECS presents a longer stability period and a longer lifetime. It is obvious from the figure that the proposed protocol offers a longer network lifetime, especially in the stability phase, in which all the nodes in the network are active. This enhancement is due to the consideration of quantum techniques, ARO, an efficient objective function, and improved quantum gates.

**Figure 7:**
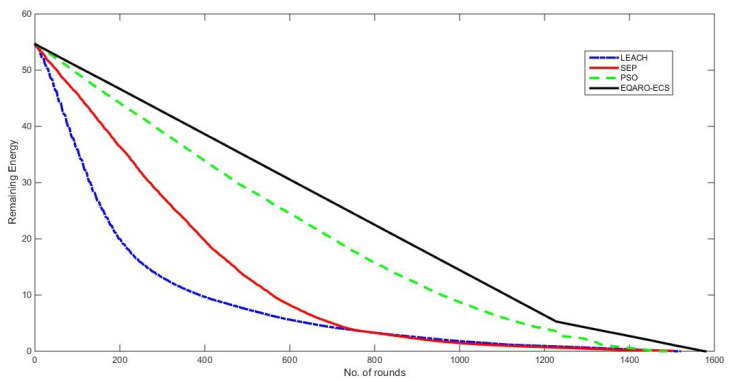
The energy depletion of EQARO-ECS, PSO, SEP, and LEACH.

Energy depletion is similarly a significant indicator of the efficiency of WSN. The best WSN routing is the network with the longest node lifetime, lowest maintenance cost, least energy depletion, and least replacement of node batteries. Figure 7 shows that the energy consumed by EQARO-ECS, for the entire nodes, is lower than that of other techniques. This is, first, due to the advantage of quantum algorithm-based gates in Equations (31) and (40) when searching for the optimal solution and, second, due to the efficient proposed fitness function presented by Equation (8).

**Figure 8:**
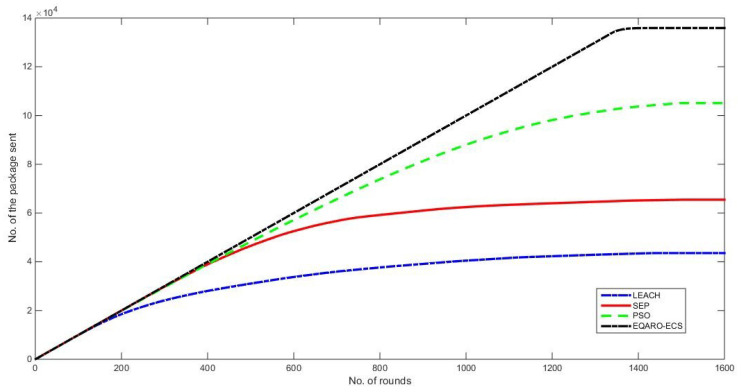
The amount of data transmitted of EQARO-ECS, PSO, SEP, and LEACH.

The EQARO-ECS improves the amount of data transmitted to the BS and then to the ECS layer in comparison to the other implemented techniques, as presented in Figure 8. This improvement is because of the growth in infrastructure lifespan and, principally, the stationary phase. The suggested EQARO-ECS technique shows an improvement in the amount of data sent of 30%, 56%, and 70% over the other AI algorithms of PSO, SEP, and LEACH, respectively.

The reason for this improvement is the yield of the implementation of Equations (1)–(3) by the presented network architecture and algorithms throughout the CT creation process. In comparison with other clusters, which use traditional techniques, clusters may result in higher energy depletion by transmitting the same amount of data as others. In conclusion, there is a positive association between the increasing quantity of data sent and the AI, objective function, and quantum techniques. All these techniques can improve network lifetime and improve the overall number of packets sent.

## 10. Conclusions

Whether the IoT protocols presented by other researchers follow traditional or AI technologies, they still need to live longer lifetimes and avoid desertification, even with environmental changes. Therefore, for the development of an appropriate algorithm, it is essential that it considers temperature and humidity to prolong the network lifespan and promise a trusted network for such an area. Whichever optimisation algorithms are used, they still easily fall into a local optimum during the cluster formulation process, and it is difficult to find the best solution with optimal energy consumption. Emerging technologies should be applied during the cluster formulation process to solve such issues.

This research proposed an AI algorithm named QARO, based on efficient technologies, which were quantum computing, ARO, EC, SDN, and Cloud for IoT networking. This algorithm improves the capability of finding the accurate global optimum and expands the network lifetime. For instance, the EC is used as a distributed computing pattern in which most or all processing is achieved on distributed nodes. A developed quantum technique within two gates (a rotation gate and an iterated T-gate) could be deployed to prevent falling into the local optimum. The simulation results showed that compared with traditional routing algorithms, such as PSO, and classical protocols, such as LEACH and SEP, this algorithm has less energy consumption and more accuracy, which demonstrates its efficiency in finding the optimal clustering technique.

Future work in IoT networking will be in the direction of merging the IoT and LoRaWAN devices with 6G and AI for smarter techniques. This case is implemented by addressing other challenges and limitations such as security (i.e., quantum methods), efficient energy techniques (i.e., energy harvesting), and reliability (i.e., bio-inheritance algorithms and designs), which enable innovative uses for smarter life applications and produce almost independent control systems.

## Figures and Tables

**Table 1:** This is the related work overview.

Category	Reference	Brief description
Classical clustering algorithms	[25,26,27,28,29,30,31,32]	Algorithm-based probability method. The main measuring unit of those methodologies is rounds. During each round, each device has a probability (*p*) of being a cluster head (CH). Accordingly, the CHs are elected based on their *p*-value. All CHs broadcast an announcement message to the adjacent devices. The non-CH devices transmit their data by electing a CH with the highest signal power. However, all the approaches have been published with a few differences
Clustering based AI and emerging technologies	[33,34,35]	Reinforcement learning (RL)-based routing protocol for WSN
	[36]	Federated learning (FL), which trains the model locally to improve efficiency and security without sharing any row data
	[37,38]	Bio-inspired techniques based on ant colony optimisation
	[5,7]	Future search algorithm (FSA)-based SDN and cloud using energy, communication cost and temperature for identifying the best set of clusters
	[39]	WOA-based SDN and cloud energy using communication, energy and node density to find best clustering
	[15,16]	GA to find the shortest path and clusters
	[40]	The protocol offered an energy-aware routing algorithm for cluster formulation based on density (EA-DBCRP)
	[41]	The authors presented a technique for forest fire discovery by developing an environmental technique for associating fault-tolerant routing algorithms that identify network reply time to an incident and network lifetime, taking into consideration network attributes
	[42]	Offered the utilisation of a quantum genetic algorithm (QGA) to elect routes among nodes and create connections for the purpose of packet exchanging
	[16,43]	Studied the implementation of quantum theory to enhance the performance of GA and evolutionary techniques
	[44]	QPSOEEC protocol is proposed, which uses a quantum-based technique for cluster formulation using a PSO
	[45]	Utilised the QPSO to find the optimal set of clusters and enhance the correctness of the node position
Proposed EQARO-ESC	EQARO-ESC	Proposes the ARO with a quantum technique, based on an EC and supported by an efficient objective function to find the best CT for IoT networking that avoids desertification factors

**Table 2:** EQARO-ECS parameters.

Symbol	Quantity	Description
*K*	40	ARO population size
$Max_{itr}$	400	Maximum number of iteration
ω	0.3	Energy parameter
β	0.3	Communication distance parameter
τ	0.4	Desertification parameter
*V*	100	number of nodes
$Eng_{elec}$	50 nJ/b	Energy dissipated to process one bit
$\xi_{TR}$	0.0013 pJ/bit/m^4^	Amplifier energy for multipath space
$\xi_{FS}$	10 pJ/b/m^2^	Amplifier energy for free space
$Eng_{DA}$	5 nJ/bit	Energy for data aggregation
$dis_{0}$	$\sqrt{\frac{\xi_{FS}}{\xi_{TR}}}$	Transmission distance threshold
Packet	4000 bit	Data message size
ρ	4	T-gate iteration times

## Data Availability

The original contributions presented in this study are included in the article. Further inquiries can be directed to the corresponding author.
